# Rho Kinases and Reactive Oxygen Species in Autophagy Regulation by Pressure in Periodontal Ligament Cells

**DOI:** 10.1590/0103-6440202405944

**Published:** 2024-12-06

**Authors:** Miriam Hardt, Alexandra Mayr, Eric Kutschera, Jana Marciniak, Erika Calvano Küchler, Christian Kirschneck, James Deschner, Andreas Jäger, Svenja Beisel-Memmert

**Affiliations:** 1 Department of Orthodontics, University Hospital Bonn, Medical Faculty, Bonn, Germany; 2 School of Dentistry, Universidade Tuiuti do Paraná, Curitiba, Brazil; 3 Department of Periodontology and Operative Dentistry, University Medical Center of the Johannes Gutenberg University, Mainz, Germany

**Keywords:** autophagy, mechanical loading, mechanotransduction, reactive oxygen species, rho-kinase

## Abstract

Autophagy is a self-digestion mechanism of cells, which is related to cell stress. It enables cell survival by maintaining cellular homeostasis or initiates cell death. This study aimed to investigate the intracellular signaling of pressure-induced autophagy regulation in human periodontal ligament (PDL) cells and to analyze the involvement of Rho kinases (ROCK) and reactive oxygen species (ROS) in particular. Human PDL cells were treated with the ROCK inhibitor Y-27632 and the ROS scavenger N-acetylcysteine (NAC) in combination with pressure magnitudes of 2, 6, and 8 g/cm^2^ over 16 hours. Cells treated with rapamycin served as a positive control and untreated cells as a control group. The Cyto-ID® Autophagy Detection Kit was used for flow cytometric analysis. Statistical analysis was performed using ANOVA and post-hoc tests. The results show that the pressure-induced autophagy was affected differently by the two inhibitors (p<0.05). The application of Y-27632 led to a significant reduction in autophagy in all pressure groups. The application of NAC led to reduced autophagy at pressures of 2 g/cm^2^ and 6 g/cm^2^. At 8 g/cm^2^, this effect was no longer present. In the control group, autophagy was significantly reduced by Y-27632 and significantly increased by NAC. Our data suggest that both Rho-kinase and reactive oxygen species could influence pressure-induced autophagy regulation in PDL cells.

## Resumo

A autofagia é um mecanismo de autodigestão das células, que está relacionado ao estresse celular. Ela permite a sobrevivência das células, mantendo a homeostase celular ou iniciando a morte celular. O objetivo deste estudo foi investigar a sinalização intracelular da regulação da autofagia induzida por pressão nas células do ligamento periodontal humano (PDL) e analisar o envolvimento de Rho kinases (ROCK) e espécies reativas de oxigênio (ROS) em particular. As células do ligamento periodontal humano foram tratadas com o inibidor de ROCK Y-27632 e o eliminador de ROS N-acetilcisteína (NAC) em combinação com magnitudes de pressão de 2, 6 e 8 g/cm2 durante 16 horas. As células tratadas com rapamicina serviram como controle positivo e as células não tratadas como grupo de controle. O kit de detecção de autofagia Cyto-ID® foi usado para análise de citometria de fluxo. A análise estatística foi realizada usando ANOVA e testes post-hoc. Os resultados mostram que a autofagia induzida por pressão foi afetada de forma diferente pelos dois inibidores (p<0,05). A aplicação do Y-27632 levou a uma redução significativa da autofagia em todos os grupos de pressão. A aplicação de NAC levou à redução da autofagia nas pressões de 2 g/cm2 e 6 g/cm2. A 8 g/cm2, esse efeito não estava mais presente. No grupo de controle, a autofagia foi significativamente reduzida pelo Y-27632 e significativamente aumentada pela NAC. Nossos dados sugerem que tanto a Rho-kinase quanto as espécies reativas de oxigênio podem influenciar a regulação da autofagia induzida pela pressão nas células PDL.

## Introduction

Autophagy is a cellular self-digestion process that can lead to both cell survival and cell death when cells are confronted with hypoxia, inflammation, nutrient deficiency, oxidative stress, mechanical stress, and other forms of cellular stress ^1^ ^,^ ^2^. In general, autophagy takes place in seven phases: initiation, nucleation, elongation, autophagosome fusion, maturation, fusion with lysosomes, and degradation of cargo ^1^. Autophagy regulation is a complex interplay of different regulators and protein complexes. Key elements of autophagy regulation are the kinase mechanistic target of rapamycin (mTOR), Unc-51 Like Autophagy Activating Kinase 1 (ULK1), adenosine monophosphate-activated protein kinase (AMPK), Beclin-1, and various autophagy-related genes (ATGs) ^3^ ^,^ ^4^ ^,^ ^5^.

Mechanical stress in the form of pressure is a strong trigger for autophagy. Mechanosensitive cells transmit external stimuli to the cell interior by mechanotransduction, where they are converted into biochemical stimuli ^1^.

During orthodontic tooth movement (OTM), mechanical forces act on cells of the periodontal ligament (PDL cells). These fibroblasts are the predominant cell population in the PDL and are originally derived from ectomesenchymal cells of the cranial neural crest. They are essential for the remodeling processes during OTM ^6^. The mechanosensitive PDL cells exhibit a tissue response through specific mechanotransduction signaling cascades ^7^ ^,^ ^8^. An important component of cellular mechanotransduction is the enzyme Rho kinase (ROCK). A study by Martino et al. ^9^ showed that mechanical signal propagation and processing can be controlled via activated Rho and the Rho/ROCK system.

Autophagy can also be triggered and regulated by oxidative stress in the form of reactive oxygen species (ROS). ROS are formed by a partial, incomplete reduction of elemental oxygen and are short-lived molecules with high reactivity ^10^. ROS can react intracellularly as second messengers to extracellular stimuli and lead to a corresponding cell response through a signaling cascade. Recent studies showed a close relationship between ROS and autophagy ^11^. In recent years, research has been conducted on the ROS-induced regulatory mechanisms of autophagy and several signaling pathways have already been identified. However, it is still unclear if ROS also plays a role in mechanically induced autophagy. This study aimed to investigate the involvement of ROCK and ROS in the pressure-induced autophagy regulation in PDL cells. We hypothesized that both ROCK and ROS are involved in mechanotransduction and subsequently influence autophagy regulation.

## Materials and methods

### Cell cultivation

To cultivate human PDL cells (Lonza, Basel, Switzerland, # CC-7049), 0.1 ml cell suspension with 10 ml Dulbecco's minimal essential medium (DMEM, Gibco, Life Technologies Limited, Paisley, UK, # 41966-029) with 10% fetal bovine serum (FBS, Invitrogen, Massachusetts, USA, # 10082147) was seeded in cell culture flasks (75 cm^2^, internal surface: Polystyrene; Greiner Bio-One GmbH, Frickenhausen, Germany; # 658175). Cells were kept at 37 °C and 5 % CO^2^. The medium was changed twice a week. Cells from the 4th to 6th passage were used for the experiments. Cells were harvested at a confluence of 80-90 %. In preparation for the experiments, cells were seeded on oxygen-permeable Lumox® culture dishes (Sarstedt, Nümbrecht, Germany; #94.6077.331). To increase cell adhesion, an attachment factor (Biologics Attachment Factor 1X, Gibco, Life Technologies Corporation, NY, USA; # S-006-100) was used. One day before the start of the experiment, the FBS concentration was reduced to 1 %.

### Cell stimulation

The PDL cells were stimulated over a constant period of 16 h with established pressure protocols ^12^ ^,^ ^13^. A physiological load of 2 g/cm^2^, a load of 6 g/cm^2^, and an overload of 8 g/cm^2^ were applied to the PDL cells in the form of glass weights (Glas Söller GmbH). Non-stimulated and unstained cells constituted the negative control group. All PDL cells, except those of the negative control, were treated with 20 µM of the autophagy inhibitor chloroquine (Enzo Life Sciences, content of the Cyto-ID® Autophagy Detection Kit, # ENZ-51031), which disrupts the autophagic flux, namely autophagosome degradation and allows autophagosomes to accumulate over time, therefore the amount of autophagosomes thus allows conclusions about autophagy. In addition, cells treated with the autophagy inducer rapamycin (50 nM; Enzo Life Sciences, content of the Cyto-ID® Autophagy Detection Kit, # ENZ-51031) served as the positive control. Experimental groups were additionally treated or not with the ROCK inhibitor Y-27632 (10 µM; Tocris Bioscience, Bristol, UK, # 1254) or the ROS scravenger N-acetylcysteine NAC (5 mM; Sigma-Aldrich, Taufkirchen, DEU, # A7250-25G). A continuous oxygen supply and constant CO^2^ removal were ensured by the use of gas-permeable Lumox® culture dishes. During the 16 h stimulation period, the cells were kept at 37°C in the incubator.

### Autophagy detection

Staining of PDL cells was performed using the Cyto-ID® Autophagy Detection Kit (Enzo Life Sciences, #ENZ-51031). Autophagy was measured with subsequent flow cytometric analysis (FACS). In brief, after the 16 h stimulation period, the glass weights were removed, the PDL cells were washed with Dulbecco’s Phosphate Buffered Saline (DPBS, Gibco, Life Technologies Limited, Paisley, UK, # 14190-094) and detached from the bottoms of the Lumox® culture dishes with trypsin. After centrifugation for 5 min, the resulting cell pellet was resuspended in 250 µl/tube Assay Buffer and the PDL cells were incubated with Cyto-ID® Green Detection Reagent (250 µl/tube) for 30 min in the dark. The stained cells were then centrifuged again, washed, and pipetted through a pluriStrainer® mini cell strainer (pluriSelect Life Science) to avoid duplicates.

### FACS

The fluorescent autophagy marker Cyto-ID® Green Detection Reagent accumulates in the autophagic vacuoles in a pH-dependent manner and thus enables measurement of the autophagy by flow cytometry. Therefore, the fluorescence can be regarded as a measure of the number of autophagosomes. The FITC filter (530/30 green) of the blue laser of a BD FACSCalibur (BD Biosciences, Franklin Lakes, USA) was used. To avoid the inclusion of duplicates and cell debris, cells were gated accordingly

### Statistical analysis

For statistics, the Prism software (Version 9, GraphPad Software, Inc., San Diego, CA, USA) was used. All experiments were performed in triplicate and reproduced at least twice. The Kruskal Wallis test followed by the post-hoc Dunnett’s test was used for statistical comparisons between groups. The significance level was set at 0.05 (p < 0,05).

## Results

### Effects of mechanical loading on autophagy

**Figure 1:**
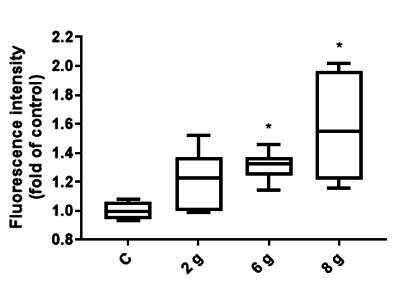
Comparison of the fluorescence intensity of the pressure groups with that of the control group. Since the dye used stains autophagosomes, the fluorescence can be regarded as a measure of the number of autophagosomes and therefore, autophagy. Pressure loads of 6 g/cm^2^ and 8 g/cm^2^ led to a significant upregulation of autophagy. No significant effect was apparent at a load of 2 g/cm^2^. Fluorescence intensity is shown as a fold of control. Median and interquartile ranges are shown (n = 144 replicates); * significant (p <0.05) difference between groups as determined by Dunnett’s T-test.

The effects of a physiological load of 2 g/cm^2^, a load of 6 g/cm^2^, and an overload of 8 g/cm^2^ on the PDL cells for 16 h were evaluated based on autophagosome accumulation using FACS analysis. A positive and negative control was included. Fluorescence and therefore autophagy was significantly elevated with increasing pressure load of 6 g/cm^2^ and 8 g/cm^2^ compared to the control group. A detailed comparison showed that autophagy at a load of 8 g/cm^2^ increased from 1 to 1.5813 and at a load of 6 g/cm^2^ to 1.3088. The adjusted P value at a load of 6 g/cm^2^ was p=0.0250 while it was p=0.0001 at a load of 8 g/cm^2^. Under a physiological load of 2 g/cm^2^, no significant effect on autophagy was apparent (Figure 1).

### Effects of Y-27632 and NAC on basal autophagy activity

**Figure 2:**
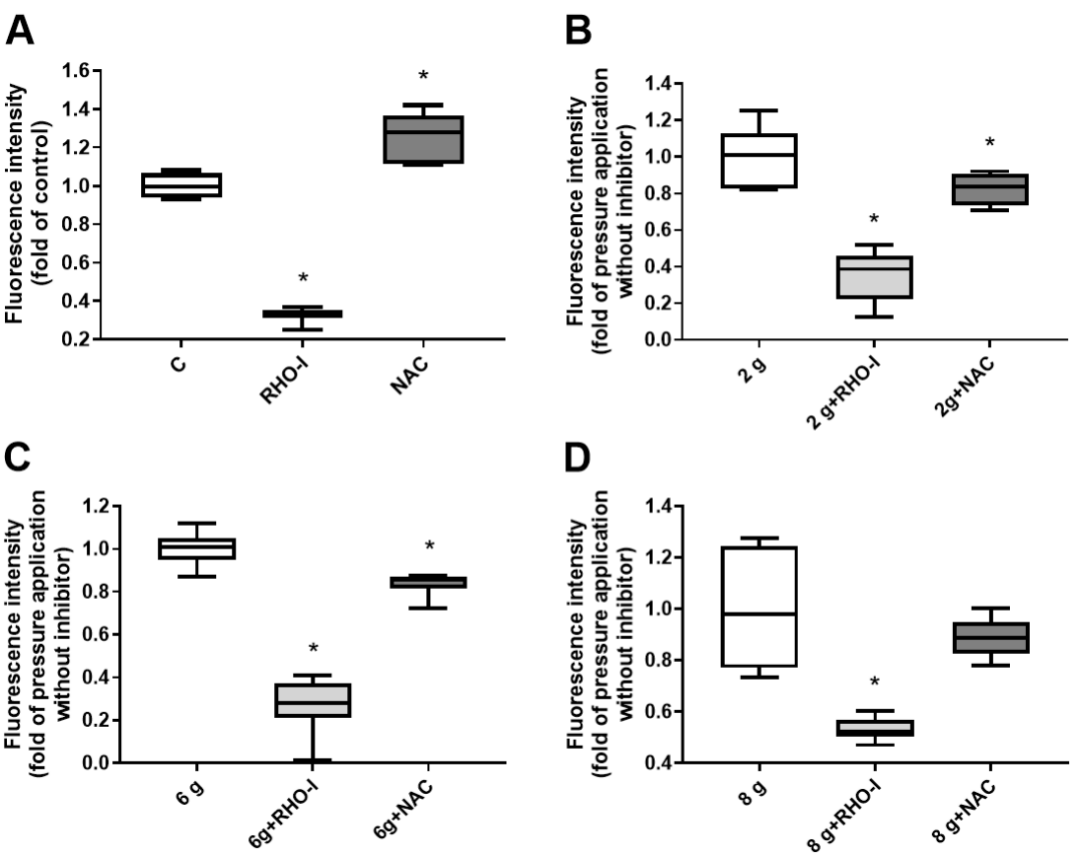
Comparison of the fluorescence intensity of the two inhibitors with the respective control group without the inhibitors. Since the dye used stains autophagosomes, the fluorescence can be regarded as a measure of the number of autophagosomes and therefore, autophagy. A) In the unstimulated group, Y-27632 reduced autophagy significantly, while NAC led to a significant upregulation. B) Inhibitor Y-27632 and NAC reduced autophagy significantly in the physiological pressure group of 2 g/cm^2^. C) Both inhibitor applications led to significantly reduced autophagy under a load of 6 g/cm^2^. D) Inhibitor Y-27632 caused a significant autophagy reduction at an overload of 8 g/cm^2^, while NAC application had no significant effect. Fluorescence intensity is shown as a fold of respective control without inhibitors. Median and interquartile ranges are shown (n = 144 replicates); * significant (p <0.05) difference between groups as determined by Dunnett’s T-test figures

The effects of the ROCK inhibitor Y-27632 and the ROS scavenger NAC were first tested on control cells to analyze how basal autophagy activity is influenced by the inhibitors. Autophagy was changed by both chemicals and, interestingly, a contrary effect was observed. The use of Y-27632 showed significantly reduced autophagy. However, treatment with NAC significantly increased autophagy. A comparison of the efficiency of both inhibitors showed a stronger effect of Y-27632 (Figure 2A). Autophagy was reduced from 1 to 0.3275 by Y-27632 and upregulated from 1 to 1.2563 by NAC. Both adjusted P-Values were p=0.0001.

### Effects of combined mechanical loading and Y-27632 on autophagy

The effects of the ROCK inhibitor Y-27632 on pressure-induced autophagy were then investigated. Under these conditions, autophagy was significantly reduced in all pressure groups, 2 g/cm^2^, 6 g/cm^2^, and 8 g/cm^2^ (Figure 2B, C, D). Interestingly, depending on the amount of mechanical load on the PDL cells, the effect of the inhibitor varied. If comparing the pressure groups with a physiological load of 2 g/cm^2^ with and without inhibitor, autophagy was reduced from 1 to 0.3503 (Figure 2B). A pressure load of 6 g/cm^2^ combined with the inhibitor resulted in a reduction of autophagy to 0.2659 (Figure 2C) compared to the pressure load alone. The comparison in the groups with the load of 8 g/cm^2^ showed that autophagy was only reduced to 0.5344 in combination with the inhibitor (Figure 2D). All adjusted P-Values were p=0.0001.

### Effects of combined mechanical loading and NAC on autophagy

The effect of the ROS inhibitor NAC on pressure-induced autophagy was also analyzed. Treatment of the PDL cells with NAC led to a significantly lower autophagy in the pressure groups of 2 g/cm^2^ and 6 g/cm^2^. Although this inhibition was still present as a trend at a load of 8 g/cm^2^, it was no longer significant (Figure 2B, C, D). Autophagy was reduced from 1 to 0.8231 and 0.8369 at a physiological load of 2 g/cm^2^ and a load of 6 g/cm^2^, respectively (Figure 2B, C). The adjusted P-value was p=0.0309 at a load of 2 g/cm^2^, p=0.0029 at a load of 6 g/cm^2,^ and p=0.2271 at an overload of 8 g/cm^2^.

## Discussion

Taken together we found strong evidence that confirmed our hypothesis that both ROCK and ROS are both involved in mechanotransduction and subsequently influence autophagy regulation. Treatment with the ROCK inhibitor Y-27632 led to significantly reduced autophagy in all pressure groups. In contrast, treatment with the ROS inhibitor NAC only led to a significant inhibition of autophagy in the pressure groups of 2 g/cm^2^ and 6 g/cm^2^, while this effect was no longer observed at 8 g/cm^2^. A comparison of the effect of the two inhibitors on the pressure-independent control group showed a contrary effect. Y-27632 led to significantly reduced autophagy, while it was significantly increased by NAC. An assessment of the results of the mechanical loading alone on the PDL cells suggests a dose-dependent autophagy regulation. Autophagy was significantly increased by pressure magnitudes of 6 g/cm^2^ and an overload of 8 g/cm^2^. No significant effect was observed with a physiological load of 2 g/cm^2^.

The presented experimental concept was based on previous studies that had already exposed PDL cells to different mechanical load magnitudes. The studies by Blawat et al. ^12^ and Schröder et al. ^14^ described 2 g/cm^2^ as a physiological pressure, which is why this force magnitude was also used in this study. In the literature, different force magnitudes have been postulated as an overload of the cells. Kanzaki et al. ^15^ described an overload with mechanical stress of 4 g/cm^2^, whereas Blawat et al. ^12^ saw a critical threshold value at 8 g/cm^2^. The different values can probably be explained by the different experimental setups. While Blawat et al. ^12^ used oxygen-permeable cell culture dishes, Kanzaki et al. ^15^ used standard cell culture plates. It can be assumed that in the experiments performed by Kanzaki et al. ^15^ both, mechanical stress and hypoxia acted as stressors and thus led to a faster overload, while in the study by Blawat et al. ^12^, the oxygen-permeable cell culture dishes led to a decoupling of the two stimuli. In the present study, the mechanical load on the PDL cells was also applied in oxygen-permeable cell culture dishes, which is why the overload of 8 g/cm^2^ was based on the study by Blawat et al. ^12^.

To focus on the involvement of ROCK and ROS in autophagy regulation, PDL cells were stimulated for a constant period of 16 h. The stress duration was based on the experimental results of Blawat et al. ^12^, where different time points such as 4 h, 16 h, and 24 h of mechanical load were investigated in PDL cells. The time point 16 h showed the highest fluorescence intensities resulting from autophagosome accumulation with the chosen set-up including the use of chloroquine and therefore disrupting the autophagic flux, which means that autophagosomes accumulate over the observed timeframe. The amount of autophagosomes is therefore an indicator of autophagy activity. In the earlier timepoints, autophagosome accumulation was less pronounced and therefore the changes in autophagy were less apparent. After 24 h, cell death increased and therefore we chose a 16 h stimulation for the present investigation.

The experiments in this study were carried out as an *in vitro* model. Although the complexity of the *in vivo* situation can never be reproduced exactly with this method, the model also offers advantages by creating a defined, differentiated situation and allowing individual parameters to be considered separately. In our experiments, it was possible to decouple mechanical stress and hypoxia by using oxygen-permeable cell culture dishes.

The present study aimed to investigate the involvement of ROCK and ROS in autophagy regulation under mechanical stress in PDL cells. The fact that mechanical stress induces a dose-dependent autophagy regulation in PDL cells has already been investigated in previous studies of our research group ^7^ ^,^ ^12^ ^,^ ^13^. The results of this work were consistent with the previously published studies.

The investigations into the influence of ROCK in cell signaling resulting in autophagy regulation under pressure were performed by ROCK inhibition. The use of the ROCK inhibitor Y-27632 clarified that the inhibition of ROCK led to a significant reduction in autophagy in all stress groups and the control group. In the study by Wang et al. ^16^, the use of Y-27632 led to increased proliferation and migration of multipotent PDL stem cells without increasing apoptosis. This provides initial evidence that the inhibitor alone does not have a cell-damaging effect. The studies by Dakic et al. ^17^, Diao and Hong ^18^, and Ichikawa et al. ^19^ even implied an anti-apoptotic effect of the inhibitor. The close relationship and interactions between autophagy and cell death have already been investigated in the literature ^9^. Memmert et al. ^7^ and Blawat et al. ^12^ showed that a variety of different signaling pathways can sequentially initiate autophagy and apoptosis in PDL cells. Thus, the inhibition of autophagy in our experiments could at least provide an explanatory model for the anti-apoptotic effect of the inhibitor. This could explain the autophagy regulation not only under pressure but also in the control group. However, as the studies investigated different cell types, such as keratinocytes, human corneal endothelial cells, and human embryonic stem cells, our study can only provide initial indications. Furthermore, the present experimental results can be compared with the study results by Mleczak et al. ^20^. In their study, ROCK involvement in autophagy modulation was also investigated in a series of experiments using human embryonic kidney cells. Like in this study, Mleczak et al. ^20^ also concluded that the ROCK signaling pathway plays a role in autophagy modulation. However, their study showed that enhanced ROCK signaling led to autophagy inhibition and inhibition of ROCK led to an enhanced autophagy response, whereas in the present work, ROCK inhibition led to reduced autophagy. These discordant results suggest that ROCK affects autophagy in human embryonic 293 cells in a different manner than in PDL cells. Our experiments are a first step to elucidate ROCK-signal transduction under mechanical load in PDL cells. The results of this study suggest ROCK involvement in autophagy-associated signaling pathways in both pressure-dependent and pressure-independent ways. In the future, it remains to be clarified if there is a threshold from which on ROCK affects autophagy independent of the mechanical load.

In addition to the involvement of ROCK in pressure-induced autophagy in PDL cells, the effect of the ROS scavenger NAC was also investigated. The aim was to provide initial information on the involvement of ROS in the induction of pressure-induced autophagy. Existing literature implies an important role of ROS in autophagy induction under different stress conditions ^21^. Our finding may complement the studies of Li et al. ^11^ and those of Scherz-Shouval and Elazar ^21^ ^,^ ^22^, as these studies, postulate a regulation of autophagy by ROS under oxidative stress, hypoxia, and nutrient deprivation. A study by Fang et al. ^23^ used ROS to induce cellular autophagy and showed that NAC blocked autophagy. Thus, the experimental results of the present work are consistent with those of Fang et al. ^23^. The study by Halasi et al. ^21^ found that NAC had a dual effect. Accordingly, NAC acted as an inhibitor of both ROS and proteasome inhibitors. The results of Halasi et al. ^24^ also showed that NAC completely abolished ROS-dependent cell death and thus the study can be reconciled with the present experimental results. In detail, the present results showed that treatment with NAC at a mechanical load of 2 g/cm^2^ and 6 g/cm^2^ led to a significant reduction in autophagy, whereas this effect was no longer observed at 8 g/cm^2^. This result can be interpreted as meaning that the inhibitory effect of NAC on autophagy is reduced at high-pressure loads and that there may be a threshold depending on pressure magnitude. A study by Luo et al. ^25^ showed that NAC treatment had a positive effect on ROS-induced reduced cell viability and cell cycle arrest. Considering the close interactions of autophagy and cell death, these results are also consistent with our findings. However, since NAC has several functions in the cell, future studies should clarify whether the influence of NAC on autophagy is based on its function as a ROS scavenger. Interestingly, NAC leads to significantly increased autophagy in PDL control cells. Therefore the basal autophagy was enhanced by the ROS scavenger. This result is in contrast to the existing literature, where NAC was found to reduce basal autophagy in African green monkey kidney cells and HeLa cells, cells that originate from human cervical carcinoma ^26^. Therefore, further studies are needed to test the effect of different ROS scavengers on PDL cells.

## Conclusion

Our data suggest that both ROCK and ROS could influence pressure-induced autophagy regulation in PDL cells. ROCK is involved in autophagy regulation not only under mechanical stress but also in a pressure-independent manner. Furthermore, the effects of ROS in autophagy activation seem to be dependent on the magnitude of applied pressure and shall be investigated in more detail in future studies.
